# A Mental Health Survey of Different Ethnic and Occupational Groups in Xinjiang, China

**DOI:** 10.3390/ijerph14010046

**Published:** 2017-01-05

**Authors:** Ailing Fu, Bo Liu, Yu Jiang, Junling Zhao, Guanghui Zhang, Jiwen Liu

**Affiliations:** 1 Department of Occupational Health and Environmental Health, College of Public Health, Xinjiang Medical University, Urumqi 830011, China; 13639949635@163.com (A.F.); Li591552613@163.com (B.L.); jiangyu@stu.xjmu.edu.cn (Y.J.); zhjl8899@sina.com (J.Z.); m13199848552@163.com (G.Z.); 2 The First Affiliated Hospital of Xinjiang Medical University, Urumqi 830011, China

**Keywords:** ethnicity, occupation, poor mental health

## Abstract

Poor mental health has become a serious social and public health-care burden. This cross-sectional study used multistage stratified cluster random sampling to gather mental health information from 11,891 adults (18–60 years) employed in various occupations categorized according to the Chinese Standard Occupational Classification. Mental health was measured by the General Health Questionnaire, and participants exceeding the cut-off score were defined as having poor mental health. The overall prevalence of poor mental health was 23.8%. The prevalence of poor mental health was significantly higher in the Han ethnic group than Kazak ethnic group and in health-care workers, teachers, and civil servants compared to manual workers. Females (odds ratios (OR) = 1.139, 95% confidence intervals (CI): 1.012–3.198) and knowledge workers (1.697, 1.097–2.962) were risk factors for poor mental health, while Kazak ethnicity (0.465, 0.466–0.937), other minority status (non-Han) (0.806, 0.205–0.987), and working ≥15 years in the same occupation (0.832, 0.532–0.932) were protective (*p* < 0.05). We concluded that the general level of mental health in Xinjiang, China, is higher in the Kazak ethnic group than the Han ethnic group. The prevalence of poor mental health is higher among knowledge workers than in manual workers due to high incidences of poor mental health in civil servants, health-care workers, and teachers.

## 1. Introduction

The World Health Organization (WHO) defines health as a status of well-being conferred by physiological, psychological, and social adaptation [1]. Thus, health is the unity of physiological and mental health. Mental health can be considered the health of an individual within a society, and in order for that society to function, good mental health among the population is a prerequisite [2]. Poor mental health has become a serious global social problem [3]. In fact, mental health (including substance abuse) accounts for as much as one fifth of the global disease burden [4,5]. Mental disorders can damage physical health and are more prevalent among specific occupational groups, reducing productivity and increasing unemployment and associated financial hardship [6].

Many studies have found that there is an association between mental health and certain occupations as well as gender, marital status, life style, and working conditions. Indeed, it has been shown that poor mental health among females is related to being divorced or widowed [7,8]. In regards to lifestyles, it is well established that in both developing and developed countries, drinking is harmful to physical and mental health [9]. Additionally, anxiety, depression, and other mental health problems are frequently related to heavy alcohol use [10,11]. Mario [12] published a report indicating that cigarette consumption was related to a poorer quality of life as well as mental health, and it has been documented that the physical and mental health of non-smokers is better than smokers [13].

In many developed countries, mental health has been found to be associated with socioeconomic status and ethnicity [14,15,16]. For example, British of African and/or Caribbean descent are more likely to suffer mental health problems compared to their white British counterparts [17]. Another study found that the Dutch tend to have better mental health than other ethnic groups, which can be partially explained by less stressful working conditions as well as religious beliefs [18,19], which have been shown to have important implications for the promotion of public mental health [18]. Previous studies have also shown that harsh psychosocial working conditions associated with certain occupational groups can lead to a variety of diseases [19] including mental health disorders [20,21] and cardiovascular disease [22]. Specifically, Hämmig [23] postulated that knowledge workers were more likely to have mental health problems compared to manual workers due to the severe psychosocial nature of their jobs. Other studies found work-related stress influenced the mental health of certain occupations, and that high levels of occupational stress were detrimental to the mental health of workers in those occupations [24,25]. Finally, compared to the general population, teachers [26], healthcare workers (especially nurses and physicians) [27,28], and civil servants [23,24] experienced higher occupational stress and worse psychosocial working conditions because of their job role and types of occupation [19].

Compared to the general population, certain occupational groups exhibit greater incidences of poor mental health [29]. While several studies, both domestic and foreign, have examined mental health problems among different occupational groups, most have focused on ethnic majorities [30,31,32] or a limited number of occupational types, such as healthcare workers, teachers, soldiers, students, pilots, and athletes [33,34,35,36,37,38,39]. Moreover, there is an increasing awareness of mental health problems, such as depression and anxiety, among certain occupational groups in China [40], but there has been no comprehensive study of poor mental health prevalence across occupational categories and ethnicity, even though ethnic groups may be a critical determinant of social and occupational status. The current study aimed to assess the overall level of mental health among different ethnic and occupational groups in Xinjiang province and to identify risk factors for poor mental health status.

## 2. Methods

### 2.1. Study Site and Population

We conducted this cross-sectional study using multistage stratified cluster random sampling from April to November 2015 in Xinjiang province, located in the northwest frontier of China and inhabited by 49 ethnic groups, such as Han, Kazak, and Uighur. In the study, a multistage stratified cluster sampling method was used to extract samples as follows. According to different geographical and economic conditions, the 14 prefectures and cities in Xinjiang were divided into four layers: prefecture-level cities; eastern Xinjiang, southern Xinjiang, and northern Xinjiang. In each layer, we took institutions as sampling frames and randomly selected 10–16 institutions from each region, respectively (there were about 250 people per institution). If the institution was picked, on-the-job staff were automatically entered into the sample size.

The initial number of participants selected was 13,010. After rejecting incomplete and incorrectly completed questionnaires, 11,891 working adults aged 18–60 years were included (Table 1), for an effective valid questionnaire response rate of 91.40%. All participants signed the consent form voluntarily.

According to the Chinese Standard Occupational Classification, teachers, medical workers, and civil servants are classified as knowledge workers, while workers, soldiers, and others are classified as manual workers. The study was approved by the ethics committee of Xinjiang Medical University.

### 2.2. The Instrument

This study used a structured questionnaire to collect basic social and demographic information, including sex, age, occupation, marital status, smoking (YES: daily or occasionally, NO: never or rarely), and drinking (YES: daily or more than 500 mL a week, NO: never or rarely). Mental health was screened using the General Health Questionnaire (GHQ), a self-reported instrument developed by David Goldberg in 1972 [41], now widely applied for screening mental health and psychiatric disorders [42], and whose reliability has been previously validated [43]. The study applied the GHQ-20 (Chinese version of GHQ-20 [43]), which is a supplementary questionnaire of GHQ-12 with better sensitivity [31]. The GHQ-20 was used to assess subjective well-being; it also identifies minor psychiatric disorders, and its excellent reliability and validity in doing so have been shown in many studies [44,45,46]. The GHQ-20 contains three scales and a total of 20 items. Items 1–9 are sub-scores of self-esteem, 10–15 are related to depression, and 16–20 deal with anxiety [43]. All 20 items are scored 0-0-1-1 (0 points for either of the first two options and 1 point for either of the latter two). The total score for mental health is the combined sub-scores of anxiety, depression, and self-esteem. The higher the total score, the lower the general mental health level. A total score of 4 or more points is considered high-risk, scores of 2–3 points are considered middle-risk, and 1 point or below is considered low-risk. In addition, participants were also classified as high-risk if any supplementary item was answered “poor”, “yes”, or “often”, regardless of total score [43]. In our study, the people at high-risk were considered as having poor mental health, while those at middle-risk and low-risk were considered as not having poor mental health.

### 2.3. Validity and Reliability

This study was conducted with strict quality control at every step. The mental health questionnaire used is considered the international general standard, a preliminary investigation was implemented before the formal investigation. The preliminary investigation was aimed at the occupational population of a university in Xinjiang, whereby 200 people were randomly selected, including teachers, officers, support workers, and others within the occupational population. We adopted a cross-sectional study method in which a questionnaire survey was conducted using GHQ-20. Participants were asked to complete the questionnaire independently and anonymously within 20 min after which all questionnaires were reviewed and encoded by specialized investigators using EpiData version 3.1 (The EpiData Association, Odense, Denmark) to establish a database. All investigators were qualified postgraduates studying at the Public Health Department of Xinjiang Medical University. In addition, 20% of entries were randomly compared against the original questionnaire to check the accuracy of the final database. The problems were then summarized for statistical analysis to analyze the reliability of the GHQ-20 using SPSS for Windows v. 17.0 (SPSS Inc., Chicago, IL, USA). In this study, we adopted the Delphi Consensus Method, whereby 18 experts in the field were interviewed using the Chinese and English version of GHQ-20 and these results were combined with the survey results in order to validate the content validity and reliability of the questionnaire. In our study, the content validity was deemed acceptable, as the Cronbach’s alpha was 0.862 and the split-half reliability was 0.751. All of the above quality control methods were applied to the formal investigation.

### 2.4. Statistical Analysis

All data was analyzed using SPSS for Windows v. 17.0 (SPSS Inc., Chicago, IL, USA). Continuous variables, such as anxiety, depression, self-esteem, and total GHQ-20 scores were expressed as $\bar{x}\pm s$. Categorical variables or rank variables were expressed as *n* (%). Continuous variables were compared among groups by independent sample *t*-tests or one-way ANOVA with Student-Newman-Keuls and least significant difference tests for pair-wise comparisons. Categorical variables were compared among groups by the chi-square test, and if there were statistically significant differences among the groups, the partition of the chi-square method was used for pair-wise comparison. The significant level was adjusted for multiple testing using the Bonferroni correction, the *p*-value for statistical significance is therefore *p* = 0.003 (0.05/17).

Binary logistic regression analysis was used to analyze the association between factors and poor mental health, those factors with *p-*value *<* 0.25 in the chi-square test were chosen to explore explanatory factors independently associated with poor mental health by backward stepwise (Wald) logistic regression analysis, odds ratios (OR) and 95% confidence intervals (95% CI) were used to express the strength of factors influencing mental health. The measure of predictive power for the regression model was the Wald test, and the Hosmer-Lemeshow test was applied to measure goodness of fit. The significance level in the logistic regression was set at 0.05. All tests were two-tailed.

## 3. Results

### 3.1. Regional Distribution of the Sample

Table 1 shows that regional sampling distribution of the 13,010 participants and the effective response rates for each region (91.40% overall).

### 3.2. The Prevalence of Poor Mental Health for Demographic and Occupational Sub-Groups

Table 2 presents the prevalence of poor mental health from the GHQ-20 for all valid responses (*n* = 11,891); the overall prevalence of poor mental health was 23.8%. We found there were statistically significant differences between sexes and among ethnicities, occupations, working year sub-groups, and marital status sub-groups (*p* < 0.003) by using chi-square test. The prevalence of poor mental health was higher in females (26.4%) than males (21.5%) (*p* < 0.003) and was highest in the Han ethnic group (25.7%) and lower in the Uyghur ethnic group (21.5%) (*p* < 0.003). The prevalence of poor mental health was higher for teachers (29.1%), health-care workers (28.7%), and civil servants (22.2%) compared to manual workers (*p* < 0.003), and was highest for those working less than five years at any occupation (47.4%) (*p* < 0.003). Those who were divorced or widowed had the highest prevalence of poor mental health (42.5%) (*p* < 0.003). Thus, shorter working years, divorced or widowed marital status, and employment in a knowledge occupation were associated with a higher prevalence of poor mental health according to the GHQ-20.

### 3.3. Comparison of GHQ-20 Total and Subscale Scores among Demographic and Occupational Sub-Groups

Table 3 displays all statistically significant differences in GHQ-20 total scores as well as self-esteem, depression, and anxiety sub-scores between sexes and smoking status groups, and among occupations and marital status groups (*p* < 0.003). Additionally, results indicated that the self-esteem, depression, and anxiety sub-scores were statistically different among the drinking group (*p* < 0.003). Total scores and all three subscale scores were higher in females than males, in the Han ethnic group compared to Uighur and Kazak ethnic groups, and knowledge workers compared to manual workers (*p* < 0.003). The divorced or widowed groups had higher total scores and lower self-esteem compared to the married and unmarried groups (*p* < 0.003). Smokers had higher self-esteem scores but lower depression, anxiety, and total scores than non-smokers, while drinkers exhibited higher anxiety scores and total scores than non-drinkers but lower depression and self-esteem scores (*p* < 0.003).

### 3.4. Risk Factors for the Poor Mental Health by Multivariable Logistic Regression Analysis

To identify independent risk factors for the poor mental health, we conducted multivariable logistic regression including sex, ethnicity, occupation, working years, and marital status as independent variables (*p* = 0.05 boundary value) (Table 4). Female sex (OR = 1.139; 95% CI: 1.012–3.198; *p* < 0.05) and knowledge work (OR = 1.697; 95% CI: 1.097–2.962; *p* < 0.05) were identified as independent risk factors. In contrast, Kazak ethnicity (OR = 0.465; 95% CI: 0.466–0.937; *p* < 0.05), other minority status (OR = 0.806; 95% CI: 0.205–0.987; *p* < 0.05), and working years ≥15 (OR = 0.832; 95% CI: 0.532–0.932; *p* < 0.05) were identified as protective factors.

The logistic regression model was statistically significant (χ^2^ = 11.95, *p* < 0.05), the model explained 48.5% of the variance in mental health status and correctly classified 81.69% of cases. This study suggests that the psychological health of the working population of Xinjiang is influenced strongly by sex, ethnicity, working years, and occupation. In general, females, members of the Han ethnic group, teachers, and healthcare workers demonstrated poorer mental health than males, ethnic minorities, and manual laborers.

## 4. Discussion

This is the first comprehensive study analyzing differences in mental health among ethnic and occupational groups in Xinjiang, China. Mental health levels were measured by the GHQ-20, with results demonstrating observable differences in regard to sex, ethnicity, occupation, and length of employment. Specifically, being female, as well as having an occupation in knowledge work, increased the risk of poor mental health. Protective factors included membership in the Kazak ethnic group or other ethnic minority, and ≥15 years at the same job. The overall prevalence of poor mental health in our study was 23.8%.

Total GHQ-20 scores were lower in the Uygur and Kazak minorities than in the majority Han ethnic group, possibly due to beliefs and religious activities that can lower the risk of mental health problems [47] and often give people hope and support when they are sick or in trouble [48]. Religious beliefs have also been shown to influence behavior and health in both simple and complex ways [49], and have been shown to have protective effects against depression and psychosis [18,50]. Additionally, greater religiousness has been shown to be mildly associated with fewer depression symptoms [50]. The mental health of manual workers was better than that of knowledge workers due to the high incidence of poor mental health in occupations such as civil servants, teachers, and healthcare workers. Long working hours and a heavy workload are known to be intrinsic properties of healthcare workers, and they are faced with many other psychosocial work hazards including high psychological demands, low support, and increasing violence in the workplace [51]; all these factors possibly enhance the risk of mental health [52]. In recent years, increasing violence in the workplace and poor working environments also increased the chronic stress levels of civil servants [52] and healthcare workers [53,54]. Additionally, teachers, whose profession entails intimate contact with children and young people [55], are known to be at an increased risk of mental health problems [56] due to the heightened job demands and work stress [57], including, for example, difficulties associated with managing classes effectively and developing supportive relationships with students [58].

The overall levels of anxiety and depression were higher in working females than males, consistent with a previous study examining occupational mental health [59]. Greater depression and anxiety in working females may arise from the dual roles played by working women, given that many may not only bear the pressures of work but also expectations of achieving a balance with family life. Indeed, such conflicts have been associated with greater susceptibility to depression in working women [60]. In contrast, workers engaged in the same occupation for a longer period reported better mental health, possibly because such workers can use their abundant work and social experience to deal with work and life pressures, thereby sustaining good mental health [61]. Married people also had lower risk of poor mental health for both sexes compared to people who divorced or widowed, which is consistent with other studies [62,63]. The majority of women who experienced divorce or who were widowed were more likely to suffer from reduced sexual frequency, depression, and anxiety [64]. The anxiety level of frequent drinkers was higher but the depression level was lower, consistent with a previous study [62]. In our study, the physical and mental health of smokers was better than non-smokers, which is in contrast to other studies [12,13]. This is possibly related to the reliability of our study (Cronbach’s alpha was 0.862) and influenced by other factors that were not noticed and should have been considered, such as social support, and economic and education level. While the sample size of this study was relatively large, a number of limitations should be considered. First, all participants completed the questionnaire independently, so the results may be corrupted by reporting and information bias, and the reliability of the GHQ-20 in our study was not high. Second, it is not known if these results can be extrapolated to other regions of China or other countries. Third, demographic and social factors not considered in this study, such as working conditions, social support, economic level, and education, may have influenced the mental health of the different ethnicities, and occupational groups. Forth, this cross-sectional study could not assess possible causes for these differences, such as the factors leading to higher levels of depression and anxiety in knowledge workers. Follow-up research should concentrate on the mechanisms by which ethnicity and occupation influence mental health. Nonetheless, we have identified occupational subgroups particularly prone to poor mental health (e.g., inexperienced female teachers) that require targeted programs and social support.

## 5. Conclusions

In conclusion, this study found statistically significant differences in the mental health levels among different ethnic and occupational subgroups in Xinjiang province, China. The overall mental health status of Uyghur and Kazak ethnic groups, independent of occupation, tended to be better than that of the Han ethnic group. Manual workers reported better mental health status than knowledge workers, primarily due to the poor mental health of civil servants, healthcare workers, and teachers.

## Figures and Tables

**Table 1:** Regional distribution of the sample.

Survey Region	Survey Sample Size		Effective Sample Size		Effective Response Rate
	*N* (Persons)	Proportion (%)	*N* (Persons)	Proportion (%)	(%)
Urumqi	2183	16.78	2152	18.10	98.58
Municipality					
Ili Kazak Autonomous Prefecture	2959	22.74	2366	19.90	79.96
Bayingolin Mongol Autonomous Prefecture	1508	11.59	1439	12.10	95.42
Hami region	1172	9.01	1072	9.02	91.47
Changji Hui Autonomous Prefecture	1009	7.76	948	7.97	93.95
Karamay City	791	6.08	711	5.98	89.89
Turpan region	1182	9.09	1092	9.18	92.39
Kashgar region	2206	16.96	2111	17.75	95.69
Total	13,010	100	11,891	100	91.40

**Table 2:** The prevalence of poor mental health among sub-groups (*n* = 11,891).

Index		Poor Mental Health		χ^2^	*p-*Value
		YES	NO		
		*n* (%)	*n* (%)		
Sex	Male	1368 (21.5)	4994 (78.5)	39.240	<0.001
	Female	1460 (26.4)	4069 (73.6)		
Ethnicity	Han	1754 (25.7)	5251 (74.3)	17.029	0.001
	Uyghur	710 ^a^ (21.5)	2593 (78.5)		
	Kazak	76 (21.2)	283 (78.8)		
	Other minority	288 (23.5)	936 (76.5)		
Occupation	Worker	826 (20.3)	3241 (79.7)	74.932	<0.001
	Teacher	554 ^e^ (29.1)	1349 (70.9)		
	Soldier	714 ^b,e^ (23.9)	2271 (76.1)		
	Civil servant	243 ^b^ (22.2)	850 (77.8)		
	Healthcare worker	345 ^c,d,e^ (28.7)	856 (71.3)		
	Others	146 ^b^ (22.7)	496 (77.3)		
Working years	<5	1025 (47.4)	1139 (52.6)	969.231	<0.001
	5–15	872 ^f^ (26.0)	2481 (74.0)		
	≥15	931 ^f^ (14.6)	5443 (85.4)		
Marital status	Unmarried	1255 (21.7)	4536 (78.3)	190.318	<0.001
	Married	1133 (22.7)	3861 (77.3)		
	Divorce, death of a Spouse	369 ^h,j,k^ (42.5)	499 (57.5)		
	Remarriage	71 ^h^ (29.8)	167 (70.2)		
Drinking	Yes	967 (24.1)	3040 (75.9)	0.409	0.523
	No	1861 (23.6)	6023 (76.4)		
Smoking	Yes	503 (23.3)	1659 (76.7)	0.390	0.532
	No	2325 (23.9)	7404 (76.1)		
Total		2828 (23.8)	9063 (76.2)		

Partition of the chi-square method results: ^a^ *p* < 0.003 compared to Han ethnicity; ^b^ *p* < 0.003 compared to teachers; ^c^ *p <* 0.003 compared to soldiers; ^d^ *p <* 0.003 compared to civil servants; ^e^ *p* < 0.003 compared to workers; ^f^ *p* < 0.003 compared to length of service <5 years; ^h^ *p* < 0.003 compared to unmarried people; ^j^ *p <* 0.003 compared to married people; and ^k^ *p <* 0.003 compared to those who were remarried.

**Table 3:** The General Health Questionnaire (GHQ)-20 scale and subscales score comparison of occupational group with different demographic characteristics.

	*n*	Self-Esteem	Anxiety	Depression	Total Score
Sex					
Male	6362	5.96 ± 1.42	0.66 ± 0.42	1.43 ± 1.13	4.07 ± 2.69
Female	5529	7.02 ± 1.57	0.87 ± 0.62	1.52 ± 1.22	5.28 ± 3.35
*t*		38.45	21.34	4.16	21.51
*p-*value		<0.001	<0.001	<0.001	<0.001
Ethnic					
Han	7005	8.13 ± 1.35	0.87 ± 0.62	1.52 ± 1.22	5.68 ± 1.92
Uyghur	3303	6.06 ± 1.22 ^a^	0.71 ± 0.31	1.21 ± 1.03 ^a^	5.28 ± 3.41 ^a^
Kazak	359	5.96 ± 1.42 ^a,c^	0.66 ± 0.42	1.43 ± 1.13 ^b,c^	4.07 ± 2.73 ^a,b,c^
Other minorities	1224	7.52 ± 1.06	0.81 ± 0.34	1.23 ± 1.02	5.42 ± 3.12
*F*		2087.29	81.63	64.73	26.77
*p-*value		<0.001	<0.001	<0.001	<0.001
Occupation					
Knowledge workers	4197	8.71 ± 1.61	0.82 ± 0.70	1.41 ± 1.08	6.23 ± 3.20
Manual workers	7694	6.06 ± 1.22	0.92 ± 0.35	1.62 ± 1.13	5.11 ± 2.69
*t*		10.12	9.37	9.86	19.36
*p-*value		<0.001	<0.001	<0.001	<0.001
Working years					
<5	2164	3.15 ± 0.31	0.86 ± 0.32	1.43 ± 1.23	5.42 ± 3.20
5–15	3353	5.21 ± 1.13 ^d^	0.72 ± 0.21	1.21 ± 1.03 ^d^	4.21 ± 2.51
≥15	6374	5.96 ± 1.42 ^d^	0.53 ± 0.32 ^d,e^	1.13 ± 1.02 ^d^	3.76 ± 2.63 ^d^
*F*		4334.3	1181.72	64.5	390.73
*p-*value		<0.001	<0.001	<0.001	<0.001
Marital					
Unmarried	5791	4.02 ± 1.57	0.66 ± 0.42	1.43 ± 1.13	3.26 ± 2.52
Married	4994	5.31 ± 1.21 ^h^	0.74 ± 0.31 ^h^	1.33 ± 1.01 ^h^	3.58 ± 2.64 ^h^
Divorce, death of a spouse	868	3.21 ± 1.02 ^f,g,h^	0.69 ± 0.11	1.17 ± 0.97 ^f,g,h^	4.07 ± 3.07 ^f,h^
Remarriage	238	4.81 ± 1.84 ^f^	0.86 ± 0.26 ^f,h^	1.53 ± 0.68	3.96 ± 2.45 ^f^
*F*		933.54	59.87	20.38	74.56
*p-*value		<0.001	<0.001	<0.001	<0.001
Drinking					
Yes	4007	5.02 ± 1.57	0.87 ± 0.67	1.53 ± 1.13	4.56 ± 2.16
No	7884	5.87 ± 1.61	0.53 ± 0.50	1.66 ± 1.32	4.50 ± 2.61
*t*		27.45	29.12	5.62	1.28
*p-*value		<0.001	<0.001	<0.001	0.21
Smoking					
Yes	2162	4.77 ± 1.53	0.52 ± 0.32	1.21 ± 1.01	4.07 ± 3.07
No	9729	3.52 ± 1.26	0.61 ± 0.41	1.64 ± 1.27	4.88 ± 3.31
*t*		36.71	10.57	15.72	10.53
*p-*value		<0.001	<0.001	<0.001	<0.001

Student-Newman-Keuls and least significant difference test results: ^a^ *p* < 0.003 compared to Han ethnicity; ^b^ *p* < 0.003 compared to Uighur ethnicity; ^c^ *p* < 0.003 compared to other ethnic groups; ^d^ *p* < 0.003 compared to length of service <5 years; ^e^ *p* < 0.003 compared to length of service 5–15 years; ^f^ *p* < 0.003 compared to married people; ^g^ *p* < 0.003 compared to those who remarried; and ^h^ *p* < 0.003 compared to those who were unmarried.

**Table 4:** Logistic regression analysis of multiple factors influencing mental health.

Influence Factor	Reference Group	Compare Group	β	S.E.	Wald	*p-*Value	OR (95% CI)
Sex	Male	Female	0.130	0.026	24.721	<0.001	1.139 (1.012, 3.198)
Ethnic	Han ethnic	Uyghur	0.836	0.224	1.646	0.292	2.307 (0.276, 2.482)
		Kazak	−0.765	0.212	11.661	<0.001	0.465 (0.466, 0.937)
		Other minorities	−0.216	0.180	7.446	0.001	0.806 (0.205, 0.987)
Working years	<5	5−15	−0.410	0.592	0.481	0.488	0.664 (0.208, 2.117)
		≥15	−0.195	0.207	0.978	0.012	0.832 (0.532, 0.932)
Occupation	Manual workers	Knowledge workers	0.529	0.335	5.641	0.001	1.697 (1.097, 2.962)
Marital status	Unmarried	Married	−0.329	0.663	0.246	0.62	0.720 (0.196, 2.642)
		Divorce, death of a spouse	0.128	0.250	0.261	0.61	1.137 (0.696, 1.853)
		Remarriage	−0.216	0.180	1.446	0.229	0.806 (0.566, 1.146)

Notes: backward stepwise (Wald) logistic regression analysis; OR = Odds ratios; 95% CI = 95% confidence intervals; the result of Hosmer-Lemeshow test is *p*-value = 0.251.
